# Visual Encoding of Social Cues Contributes to Moral Reasoning in Autism Spectrum Disorder: An Eye-Tracking Study

**DOI:** 10.3389/fnhum.2018.00409

**Published:** 2018-10-15

**Authors:** Mathieu Garon, Baudouin Forgeot d’Arc, Marie M. Lavallée, Evelyn V. Estay, Miriam H. Beauchamp

**Affiliations:** ^1^Department of Psychology, University of Montreal, Montreal, QC, Canada; ^2^Rivière-des-Prairies Hospital, Montreal, QC, Canada; ^3^Centro de Desarrollo de Tecnologías de Inclusión, Escuela de Psicología, Pontificia Universidad Católica de Chile, Santiago, Chile; ^4^Sainte-Justine Hospital Research Center, Montreal, QC, Canada

**Keywords:** moral reasoning, moral decision-making, visual encoding, eye-tracking, pupillometry, autism spectrum disorder

## Abstract

Eye-tracking studies suggest that visual encoding is important for social processes such as socio-moral reasoning. Alterations to the visual encoding of faces, for example, have been linked to the social phenotype of autism spectrum disorders (ASDs) and are associated with social and communication impairments. Yet, people with ASD often perform similarly to neurotypical participants on measures of moral reasoning, supporting the hypothesis of differential mechanisms of moral reasoning in ASD. The objective of this study was to document visual encoding and moral reasoning in ASD and neurotypical individuals using a visual, ecological, sociomoral reasoning paradigm paired with eye-tracking. Two groups (ASD, Control) matched for age and IQ completed the SoMoral task, a set of picture situations describing everyday moral dilemmas, while their eye movements and pupil dilation were recorded. Moral understanding, decision-making, and justification were recorded. Participants with ASD presented a longer time to first fixation on faces. They also understood fewer dilemmas and produced fewer socially adaptive responses. Despite a similar average level of moral maturity, the justifications produced by participants with ASD were not distributed in the same way as the neurotypical participants. Visual encoding was a significant predictor of moral decision-making and moral justification for both groups. The results are discussed in the context of alternative mechanisms of moral reasoning in ASD.

## Introduction

Moral reasoning has repercussions on the way individuals make decisions and behave socially and therefore is a key socio-cognitive component of everyday interactions (Krebs et al., 1997; Gold et al., 2015; Villegas de Posada and Vargas-Trujillo, 2015). While it relies on rapid and automatic mechanisms such as attentional processes, moral reasoning can also involve elaborate, deliberative reasoning (Kahneman and Sunstein, 2005; Saunders, 2009; Fiedler and Glöckner, 2015). Theoretical models suggest that, like most socio-cognitive functions, moral reasoning includes a perceptual encoding stage that is required to extract relevant information from a social situation (Crick and Dodge, 1994; Arsenio and Lemerise, 2004). Empirical studies have used eye-tracking to establish a relation between eye movements and moral decision-making (Pärnamets, 2008; Kastner, 2010; Skulmowski et al., 2014). Using variations of the trolley problem (i.e., A trolley is running on a track on which are five workers. Participants must choose between pulling a lever to lead the trolley down a sidetrack on which only one man is working, or let the trolley run its course.), these studies demonstrate that visual encoding is biased toward the chosen option and can thus predict moral decision-making. Autism spectrum disorders (ASDs) are characterized by alterations in social functioning including possible changes in moral reasoning (Moran et al., 2011; Zalla and Leboyer, 2011; Zalla et al., 2011; Gleichgerrcht et al., 2012; Margoni and Surian, 2016) and reliance on distinct social information encoding strategies when compared to neurotypical controls (Klin et al., 2002; Pelphrey et al., 2002; Jones et al., 2008; Kliemann et al., 2010; Pierce et al., 2016). We suggest that these two aspects (encoding and moral reasoning) are connected in ASD. The present study aimed to quantify this putative association.

### Autism Spectrum Disorders and Visual Encoding

Autism spectrum disorder is associated with particularities in the visual encoding of social information (Klin et al., 2002). For example, people with ASD generally make shorter (Pelphrey et al., 2002) and later (Sasson et al., 2007) visual fixations on faces, particularly in the eye region (Kliemann et al., 2010; Tanaka and Sung, 2016; Wei and Ziqin, 2018). These differences have an impact on higher-level social cognition by limiting the information available for social reasoning. Higher intensity of autistic traits in neurotypical individuals is also associated with a decrease in the frequency of gaze fixations toward the speaker during a discussion (Freeth et al., 2013), and with a decrease in the number of fixations toward faces (especially toward the eyes) when viewing social scenes (Debbané et al., 2010).

The relation between visual encoding, social cognition, and social adjustment is also well-documented in ASD and these processes are directly related to the intensity of autistic symptoms. For instance, reduced fixation time on the eyes correlates with symptoms of social anxiety in ASD (Corden et al., 2008). Similarly, decreased duration of gaze fixation toward social scenes (Chawarska et al., 2012) and fixation on the eye area (Jones et al., 2008) are associated with greater social impairments (Lord et al., 2008). Differences in visual encoding in ASD are observable in the early stages of information processing. For instance, ASD is associated with decreased emotion recognition in the range of microexpressions (15 to 30 ms) (Clark et al., 2008), possibly reflected alterations in automatic social information processes (Chevallier et al., 2013). Since encoding and processing of relevant visual information is inherent to social adaptation (Arsenio and Lemerise, 2004) and moral decision-making (Pärnamets, 2008; Kastner, 2010; Skulmowski et al., 2014; Fiedler and Glöckner, 2015), visual encoding differences in people with ASD are likely to be associated with differences in moral reasoning. However, this has never been empirically demonstrated.

### Autism Spectrum Disorders and Moral Reasoning

Deficits in social interactions in ASD are far from complete or homogeneous. In those with average intellectual functioning, difficulties in social interactions are often the predominant cause of disability and distress (Vickerstaff et al., 2007; White and Roberson-Nay, 2009; Lasgaard et al., 2010). ASD is also characterized by alterations in aspects of social information processing including encoding (Klin et al., 2002; Pelphrey et al., 2002), representation (Baron-Cohen et al., 1985; Frith et al., 1991), and social motivation (Chevallier et al., 2012; Kohls et al., 2012), which are likely to contribute to the autistic phenotype. There is no actual consensus on a cohesive explanatory model of moral reasoning in ASD. Some empirical studies suggest that several aspects of moral reasoning – namely, *moral understanding, moral judgment, moral decision-making*, and *moral justification* – are intact in ASD (e.g., James and Blair, 1996; Grant et al., 2005), while others highlighted small but significant differences, as discussed below.

Prior studies report that *moral understanding* is mostly preserved in autism (James and Blair, 1996; Grant et al., 2005). As such, individuals with ASD are able to distinguish an action affecting the well-being or the rights of another person (moral) from an action affecting social order (conventional). However, differences in moral understanding have been identified in a more recent study. Zalla et al. (2011) asked participants to evaluate transgressions of moral, conventional and hygiene rules. Despite similar results otherwise, ASD participants failed to distinguish between moral and hygiene transgressions. These findings suggest that precise control of the moral dimensions evaluated can facilitate the detection of differences that would otherwise go unnoticed.

Similarities between ASD and control participants have also been observed in *moral judgment* tasks (i.e., tasks in which participants assess the morality of others’ actions by deciding if it was blameworthy or not). Both individuals with ASD and neurotypical controls are more likely to condemn harm to a person than to material property and to judge the morality of an action by the actor’s intention rather than by the outcome (Grant et al., 2005; Leslie et al., 2006). However, those with ASD may rely on different mechanisms to achieve the same results as typically developing peers on tasks measuring moral understanding and judgment (De Vignemont and Frith, 2008; McGeer, 2008; Barnes et al., 2009; Brewer et al., 2015). Also, studies report a tendency in individuals with ASD to rely more on the outcomes of an action than on the intentions of an agent when producing moral judgments (Moran et al., 2011; Zalla and Leboyer, 2011; Margoni and Surian, 2016).

A limited number of studies have focused on *moral decision-making* in individuals with ASD. In contrast to studies focusing on moral judgment, those that test moral decision-making use first-person scenarios in which participants are asked what they would do if faced with a moral dilemma. Some have found no differences in the type of moral decisions made by individuals with high ASD traits (Vyas et al., 2017), or those with formal ASD diagnoses, and typically developing individuals (Patil et al., 2016). However, Gleichgerrcht et al. (2012) demonstrated that ASD is associated with a greater proportion of utilitarian responses (i.e., that aim to maximize well-being for the greatest number of people) on moral dilemmas, even if ASD and control participants have a similar understanding of moral issues and conceptions of right and wrong. This distinction between moral *knowledge* and moral *decision-making* is important because it suggests that asking participants with ASD about their theoretical knowledge of right and wrong or about their judgment of others’ actions is most likely not indicative of the decisions they would make in real-life situations.

To our knowledge, no studies have objectively addressed the type of justifications (*moral reasoning*) produced by individuals with ASD when asked to justify their own decisions. The cognitive-developmental approach to moral reasoning suggests that the justifications provided to support a moral decision can be quantified as consecutive levels (Kohlberg, 1981; Gibbs, 2013). For example, not stealing could be justified by the fear of being punished (low level of moral maturity) or by the universal principle of right to property (high level of moral maturity). Some studies of moral judgment have shown differences in the justifications produced by individuals with ASD when judging others’ actions. Even when they produced moral judgments comparable to those of neurotypical participants, their justifications were often less elaborate (Grant et al., 2005). They also evoked fewer abstract moral rules to justify their judgment and produced more non-specific condemnations such as “because it is wrong” (Shulman et al., 2012). Differences in the nature or level of moral justifications in ASD are plausible, given, for example, dampened attention to the social aspect of situations (Chevallier et al., 2012, 2015) and greater focus on rules and order (McGeer, 2008), when compared to typically developing individuals, but this has not been explicitly demonstrated.

### Methodological Approaches for Measuring Moral Processes

The Socio-Moral Reasoning Aptitude Level Task (SoMoral; Dooley et al., 2010; Beauchamp and Dooley, 2012; Beauchamp et al., 2013; Vera-Estay et al., 2015, 2016; Chiasson et al., 2017) is a visual moral reasoning measure that contains moral dilemmas from everyday life situations presented using pictures. It measures three aspects of moral reasoning: understanding, decision-making, and justification. The task addresses some of the methodological limitations of traditional moral reasoning measures. For example, the utility of some traditional tasks is limited when addressing questions about everyday moral reasoning. The type of stimuli used (written stories, simple cartoons) and the situations depicted (life and death scenarios, dilemmas unlikely to happen in real life, Vyas et al., 2017) limit ecological validity (Kahane, 2015). Confounding variables, such as reading skills may also be problematic, as written scenarios may favor individuals with ASD in whom verbal abilities surpass non-verbal abilities (Chiang et al., 2014). These methodological issues are particularly problematic in the study of ASD because the visual encoding differences observed in ASD are dependent on the context and nature of the social stimuli. For example, visual encoding differences between ASD participants and neurotypical controls tend to be more apparent when measured using clear dyadic signs (Chawarska et al., 2012) or ecological social interactions (Chevallier et al., 2015), whereas studies using gray scale, static stimuli fail to find visual fixation differences between ASD and neurotypical participants (Van Der Geest et al., 2002; Sterling et al., 2008; McPartland et al., 2011). When used in the context of neurotypical development, predictors of SoMoral scores include cognitive variables (e.g., executive functions) and demographic variables such as socioeconomic status and age (Vera-Estay et al., 2015, 2016). The task has also been used in clinical populations and results highlight the impact of conditions such as traumatic brain injuries (Dooley et al., 2010; Beauchamp et al., 2013) and focal brain insults (Chiasson et al., 2017) on moral reasoning. A recent study in neurotypical adults combined the SoMoral task with eye-tracking measures (Garon et al., 2018). The results suggest that visual encoding, specifically, the number of fixations produced toward social cues, is a predictor of moral justification level. It is therefore likely that this task will help characterize everyday moral reasoning and visual encoding in people with ASD.

### Aims and Hypotheses

The main objective of this study was to document and quantify the relation between the encoding of visual information and moral reasoning in ASD and to answer the following questions. Relative to typically developing controls: (1) Do participants with ASD present differences in moral reasoning, including understanding, decision-making and justification? We anticipated differences in moral reasoning with participants with ASD presenting poorer understanding of visual, ecological, socio-moral dilemmas, fewer socially adaptive responses, more justifications relying on obedience to authority and social rules, and fewer justifications oriented toward interpersonal relationships. (2) Do participants with ASD present differences in visual encoding? We expected visual encoding to be different across groups. Neurotypical participants were expected to exhibit a bias toward social cues (i.e., faces) leading to a faster detection and an increased fixation duration on these cues. This bias was expected to be reduced, or absent, among participants with ASD. (3) Is the relation between visual encoding and moral reasoning in ASD similar to the relation in typical individuals? We anticipated that visual encoding of social cues (e.g., the duration of visual fixations toward faces) would predict moral reasoning (understanding, decision-making, and justification).

## Materials and Methods

### Design

This study was a confirmatory correlational study. Scores on a moral reasoning task (understanding of dilemmas, moral decision-making score, and moral justification score) are the dependent variables. Eye movements (Time to First Fixation and Fixation Count on faces), pupillary dilation and group (Participants with ASD and comparable controls) are the independent variables.

### Participants

The experimental group consisted of 30 young adults with ASD (seven women, 23.3%) aged 17 to 34 years (*M* = 24.41, *SD* = 4.71 years). For inclusion, participants in the clinical group had to be diagnosed with ASD according to standardized instruments (ADI-R, ADOS-G) and according to DSM-IV criteria (Association American Psychiatric [APA], 2013). Assessment and diagnoses were performed by experienced clinicians. The control group consisted of 59 young adults (26 women, 44,1%) aged 16 to 44 years (*M* = 22.80, *SD* = 6.39 years) with no psychiatric, developmental, or neurological disorders. Participants in the control group underwent a semi-structured interview in order to exclude any participants with a history of psychiatric treatment or learning disability. In both groups, individuals with a diagnosis of epilepsy, schizophrenia or intellectual disability were excluded, as were those with motor or sensory problems likely to interfere with the experiment. All participants spoke French. All participants provided informed written consent for the study. The study was approved by the local research ethic committee. All participants provided informed written consent for the study. The study was approved by the University of Montreal Research Ethics Committee [Comité d’éthique de la recherche en arts et en sciences (CERAS)]. The approval number is CEìRFAS- 2011-12-016-P.

### Material

#### Moral Understanding, Decision-Making, and Justification

**FIGURE 1:**
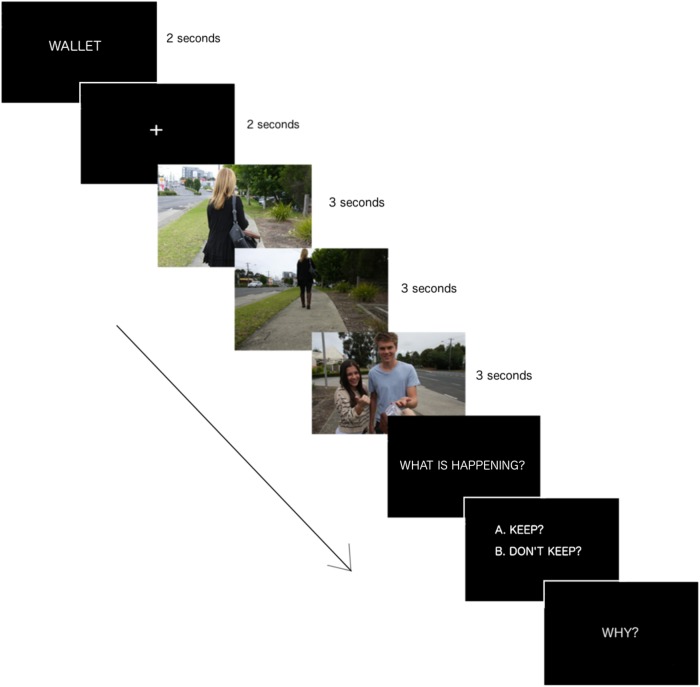
Item from the SoMoral task. The introductory screen presents the name of the dilemma. A fixation cross is then presented for 2 s. A social situation involving a moral dilemma is then presented via three first-person perspective pictures (e.g., A woman is walking and her wallet is about to fall out of her handbag (picture 1); the wallet falls out on the sidewalk while the woman continues on her way (picture 2); the participant finds the wallet and his/her friends are happy to see the money suggesting they should keep it (picture 3). The following screens present the moral understanding question, then a dichotomous decision choice and in the final screen participants are then asked to provide a justification for their decision, which is recorded verbatim for subsequent coding.

Participants completed the SoMoral (Dooley et al., 2010; Beauchamp and Dooley, 2012; Beauchamp et al., 2013; Vera-Estay et al., 2015, 2016; Chiasson et al., 2017). The SoMoral is a self-paced, visual, computer-based task. The version used presents 16 moral dilemmas, each dilemma (**Figure 1**) consisting of an introductory screen presenting a title (e.g., ‘wallet’), three first-person perspective pictures of actors playing out various social scenarios representing a moral conflict (e.g., concerns with justice, welfare/harm, and rights) according to Social Domain Theory (Turiel, 1983). The dilemmas include situations likely to occur in everyday life (e.g., a classmate asks for the answers during an exam and the participant must decide whether or not to give their answer; the participant has the opportunity to cheat while playing a board game and is asked if they would or not; the participant accidentally breaks the windshield of a car window and is asked if they would tell the owner of the car or not). Each picture was presented for 3 s and each scenario was preceded by a fixation cross. After viewing the three pictures, the participant is asked to say what they understood of the dilemma at play in the situation, to indicate what they would do if faced with the situation and to provide a justification. The three questions asked to respectively assess moral understanding, decision-making, and maturity are “What is happening?” “What would you do?” and “Why?” When the participant provide an answer to the first question (what is happening?), moral understanding is determined by comparing the response provided to pre-established criteria. These criteria are elements that must be included in participants’ responses to show that they have understood the situation. For example, for the dilemma shown in **Figure 1** (wallet), an adequate answer must mention three elements: the wallet has been lost/dropped, the participant has found it/picked it up, and their friends want them to keep it. The moral understanding score for each dilemma is 1 (understood) or 0 (misunderstood). A total moral understanding score, ranging form 0 to 16 points, is then derived for each participant. Another screen presenting a dichotomous decision (e.g., whether or not to engage in a particular action such as stealing from a shop, cheating during a game, intimidating a classmate, helping a friend, etc.) is then presented. The aggregate number of socially adaptive responses is compiled to obtain a moral decision-making score, which ranges from 0 to 16 points. Finally, participants are asked to provide a justification for their decision. Each participant’s justification is recorded verbatim and scored using a standardized coding system (Beauchamp and Dooley, 2012) based on the cognitive-developmental approach to moral reasoning (Gibbs, 2013). Developmental levels of moral reasoning have been updated and adapted to fit the social nature of the dilemmas in the SoMoral task and consist of the following: (1) Centrations and authoritarian-based consequences; (2) Egocentric/pragmatic exchanges; (3) Interpersonal focus; (4) Societal regulation; and (5) Societal evaluation. Detailed description of coding levels and examples are provided in previous articles (Vera-Estay et al., 2015; Chiasson et al., 2017). Transition levels (1.5 and 2.5, etc.) are used to account for answers that provide elements of two consecutive reasoning levels. When elements of non-consecutive levels are provided, the response is coded according to the highest schema detected. The moral justification score (0 to 80 points) is obtained by summing the 16 justification scores. This test has adequate construct validity (Dooley et al., 2010). Two trained raters scored the justifications independently. The inter-rater reliability for a proportion of the justifications (10%) was Kappa = 0.82 (*p* < 0.001), 95% *CI* (0.75, 0.89), which can be interpreted as an “almost perfect agreement” (Landis and Koch, 1977). The moral understanding, moral decision-making and moral justification scores were used as the main dependent variables.

#### Cognitive and Affective Measures

The following measures were used to control for possible confounding variables because they have been shown to be associated with social cognition in general, and moral reasoning in particular (Latif, 2000; Langdon et al., 2011; Gleichgerrcht and Young, 2013; Patil and Silani, 2014).

##### Wechsler abbreviated scale of intelligence (Wechsler, 1999)

The WASI provides an estimate of the intellectual quotient based on two sub-tests of the Wechsler scales: Matrix reasoning and Vocabulary. An estimate of the full-scale IQ (*M* = 100, *SD* = 15) was obtained for each participant and used to ensure participants compatibility.

##### Interpersonal Reactivity Index (IRI, Davis, 1983)

This 28 item self-report questionnaire addresses the construct of empathy multidimensionally by providing both an affective and cognitive (theory of mind) empathy subscale (Rogers et al., 2007). Items are scored using a Likert-type scale ranging from one to five. Subscores are generated for four subscales: Fantasy, Perspective taking, Empathic concern, and Personal distress. Fantasy is defined as the tendency to identify with characters from fiction work (e.g., movie and book). Perspective taking, the cognitive component of empathy, is described as the ability and tendency to adopt someone else’s perspective. Empathic concern represents the extent to which someone tends to be concerned for other’s well-being. Personal distress represents the emotional component of empathy and the tendency to feel discomfort or anxiety when observing someone else feeling negative emotions. The IRI factor structure is well-documented (Spraggins, 1987) and the measure has good internal consistency (alphas 0.68 to 0.79, Davis, 1980; Christopher et al., 1993). Each of the IRI subscales also have good test–retest reliability with correlation coefficients ranging from 0.61 to 0.81 (Davis, 1980). Furthermore, the IRI correlates with other measures of empathy, supporting its construct validity (Davis, 1980). Scores for all four subscales were used in the statistical analyses: Fantasy (IRI-F), Perspective taking (IRI-PT), Empathic concern (IRI-EC), and Personal distress (IRI-PD).

##### Toronto Alexithymia Scale (TAS, Bagby et al., 1994)

This is a self-report questionnaire composed of 20 items using a Likert-type scale ranging from 1 to 5. It measures participants’ ability to identify, understand, describe, and communicate the emotions they feel. A low alexithymia score indicates a better understanding of one’s emotions. The TAS provides scores for three subscales: Difficulty Describing Feelings, Difficulty Identifying Feeling and Externally-Oriented Thinking. Each of these factors has adequate internal consistency with alphas of 0.78, 0.75, and 0.66, respectively. The test–retest reliability for the full scale is 0.77 (Bagby et al., 1994). The French translation (Loas et al., 1995) was used in the current study [Cronbach’s alpha = 0.79, correlations between items and the total score ranges from 0.79 (*p* < 0.05) and 0.69 (*p* < 0.007) with a mean of 0.52].

##### Social Desirability Scale (SDS-17, Stöber, 2001)

Given the likelihood that participants respond favorably on measures of social skills to please the examiner (Schonfeld et al., 2005), participants also completed the SDS-17. The SDS-17 scale is composed of 17 dichotomous questions (true or false) measuring the extent to which participants tend to present socially positive images of themselves. The scale is similar to Crowne and Marlowe’s social desirability scale (Crowne and Marlowe, 1960), but includes more contemporary content (Blake et al., 2006). A total social desirability score was assigned to each participant. The questionnaire has adequate convergent validity (Blake et al., 2006), correlating between 0.52 and 0.85 with other commonly used measures of social desirability (Eysenck Personality Questionnaire-Lie Scale, Marlowe-Crowne Scale) (Stöber, 2001).

#### Eye-tracking Apparatus

Eye movements and pupil dilation were recorded using a Tobii T60XL eye-tracker during the SoMoral task. The eye-tracker has a sampling rate of 60 Hz, an accuracy of 0.5 degrees, and a spatial resolution of 0.35 degrees. The stimuli were presented on the custom Tobii 24-inch screen with a resolution of 1920 × 1080 pixels. Participants were positioned at a distance of 60 cm from the screen. A chin rest was used to limit participants’ head movements during the experiment.

#### Eye-Tracking Measures

**FIGURE 2:**
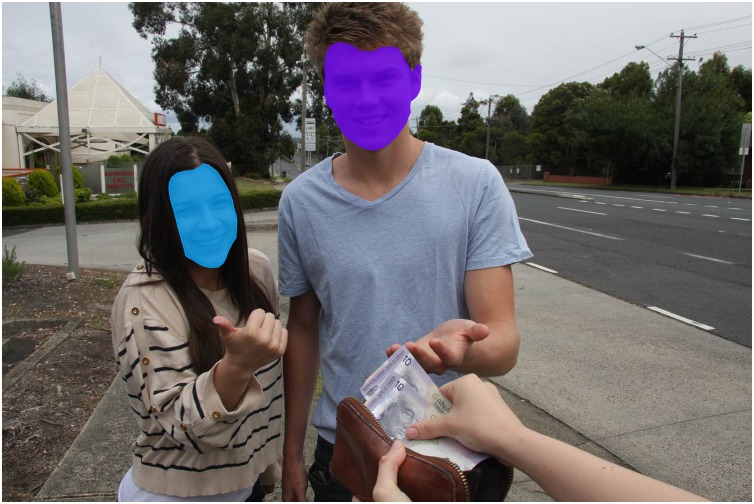
Example of selected regions of interest (ROIs).

As primary social cues, the faces of the characters contained in the moral dilemmas were defined *a priori* as regions of interest (ROI, see **Figure 2** for an example). For each picture used in the experiment, ROIs were delimited manually using Tobii’s eye-tracking software (Tobii Studio 3.2). The size of each ROI was measured in pixels and used as a covariate for statistical analyses.

The fixations were identified using the Velocity-Threshold Identification (I-VT) fixation classification algorithm (Olsen, 2012). Two metrics were extracted from the gaze data: *Time to First Fixation and Fixation Count*. *Time to First Fixation* represents the amount of time elapsed between the onset of each picture and the production of a first fixation on a ROI. In the case where several faces were present in a picture, the shorter *Time to First Fixation* was used. *Fixation Count* represents the total number of fixations produced within a ROI. When a picture contained several faces, *Fixation Counts* for every face were summed to obtain a total score. Metrics were calculated individually for the three pictures presented in each moral dilemma. For every dilemma, *Time to First Fixation* were then averaged to obtain one score for each dilemma. For *Fixation Count*, the measures for the three pictures were summed.

Pupil diameter was measured continuously during every dilemma. For each participant, a segment including the presentation of the three pictures was extracted for every dilemma. Raw data were then processed according to the procedure developed by Jackson and Sirois (2009). A digital low-pass filter with a sample frequency to cut frequency ratio of 12.5 was initially applied to the raw data in order to reduce noise and variability inherent to this type of measurement. The filter was applied twice (once forward and once backward) to ensure that processing did not cause any phase shift in the signal. Missing data (attributable to eye blink, measurement error, or to the participant looking away from the screen) were then interpolated. As pupil diameter from both eyes is highly correlated (Jackson and Sirois, 2009), when samples from a single eye were missing, the samples from the other eye were used for the interpolation. Linear interpolation was then conducted using the average value of the three samples preceding and following the break. The data for the left eye and for the right eye were then combined to obtain a single average signal on which the analyses were conducted. Overall, this procedure accounts for missing data and reduces noise, while preserving the dynamic properties of the signal for each trial. For each segment, the average pupillary diameters for the complete stimuli onset were calculated.

### Procedure

The testing session took place in a quiet, adapted assessment room over a 2-h period. Participants received financial compensation for their participation and travel. Participants first read and signed the consent form, performed the moral reasoning task and then completed the questionnaires (IRI, TAS, SDS-17) and the WASI. To ensure adequate measurement, the eye-tracker was calibrated before each participant (the participant is asked to follow a light point moving on the screen). Participants then performed the SoMoral task while eye movements were measured. They provided their answers orally and these were recorded verbatim.

## Statistical Analyses

All statistical analyses were conducted using IBM SPSS Statistics 21. Data were examined for violations of the assumption of normality before all analyses: Kolmogorov–Smirnov tests were non-significant (*ps* > 0.05) for eye-tracking variables (TTFF, FC, Pupil dilation), moral reasoning variables (understanding, decision-making, justification), and control variables (IQ, IRI subscores, SDS-17, TAS). Kurtosis and skewness distributions were all between -2 and 2, which is considered acceptable (George and Mallery, 2010). Data were also inspected for multicollinearity. For the independent variables (TTFF, FC, pupil dilation, ROI size), the variance inflation factors (VIF) were all < 2 and the condition indexes were all < 30, which is appropriate and allows the predictors to be included in the same model (Ringle et al., 2015). As the experimental design involved repeated observations (i.e., multiple dilemmas), mixed models were adjusted to determine the effect of eye movements on moral reasoning while accounting for the non-independence between the measures of the 16 dilemmas for the same subject. A random intercept model was tested and a compound symmetry covariance structure was used. A first set of analyses was conducted to ensure comparability of groups and to identify any potential confounding variables of interest. Participants in the ASD group were compared to those in the control group with respect to sex using a Chi-squared test and with respect to age, IQ, empathy (subscores of the IRI), social desirability (SDS-17), and alexithymia (TAS) using independent samples *t*-tests. Any variable for which a group difference was found was subsequently tested to verify whether it accounted for variance in any of the three SoMoral scores using mixed binary logistic regressions (Understanding, Decision-Making) or mixed linear regressions (Justification). Any variable that accounted for variance in any of the SoMoral scores was included as a covariate in the main analyses.

To address the first and second research questions, groups (control and ASD) were compared on the three SoMoral variables: Understanding (total number of dilemmas understood), Decision-making (total number of adaptive responses), and Justification (total justification score) and on eye-tracking measures (TTFF, FC, and Pupil dilation) using independent sample *t*-tests. Of note, all subsequent analyses involving moral Decision-Making or Justification were conducted only on the dilemmas that were rated as “understood” to ensure that the results were attributable to moral processes rather than to underlying cognitive or perceptual difficulties. To assess possible group differences in the types of answers provided by participants for each justification level individually, Mann–Whitney tests were also conducted (Office of Planning Assessment Research and Quality [OPARQ], 2015).

To address the third research question, binary logistic regressions were conducted using the Generalized linear mixed model procedure (GENLINMIXED) with eye-tracking variables (TTFF, FC, Pupil dilation), group (ASD, control) as predictors, and moral Understanding and moral Decision-making as outcomes. The interaction between eye-tracking measures and group (Group^∗^TTFF, Group^∗^FC, Group^∗^Pupil dilation) were also included in the models to test whether the effect of encoding on moral reasoning was different across groups. A random intercept model was tested and a compound symmetry covariance structure was used. To ensure that the putative relation between eye movements and moral reasoning were attributable to visual encoding strategies and not to stimuli properties, the size of the social cues (faces) contained in the pictures was systematically added as a covariate (ROI size in pixels) on the analyses that included eye movements’ measures.

The relation between eye-tracking variables and moral Justification score was assessed using the Linear mixed model procedure (MIXED). Eye-tracking measures (TTFF, FC, Pupil dilation), group (ASD, Control) and interaction (Group^∗^TTFF, Group^∗^FC, Group^∗^Pupil dilation) were entered as independent variables (fixed factors) and moral Justification level for every dilemma individually as a dependent outcome. As for moral Understanding and Decision-making, models included ROI sizes as a covariate.

## Results

### Group Comparisons and Confounding Variables

There were no age, IQ, or sex differences between groups (**Table 1**). Social desirability was found to be significantly higher in ASD than in control participants. The score for the Perspective-Taking subscale of the Interpersonal Reactivity Index was lower in ASD than in control participants. Otherwise, both groups were comparable on IRI Personal Distress, Fantasy, Empathic Concern, and Alexithymia (**Table 1**). Subsequent analyses found that neither IRIpt nor SDS-17 scores were significant predictors of moral understanding, decision-making, or justification (**Table 2**), and thus they were not included as predictors in the final models.

**Table 1:** Comparison of control and ASD groups on control variables using independant sample *t*-tests and chi-square test.

	Control	ASD		
			
	M (SD)	M (SD)	*t*/χ^2^	*p*
Age	22.80 (6,39)	24.41 (4.71)	−1.21	0.230
Sex	23.3% women	44.1% women	3.67	0.056
IQ	108.40 (9.55)	106.19 (10.44)	0.85	0.399
SDS-17	6.76 (3.04)	10.18 (2.58)	−4.15	<0.001^∗^
IRIpt	19.44 (5.13)	16.69 (3.35)	2.62	0.010^∗^
IRIpd	11.76 (5.70)	13.31 (5.34)	−1.22	0.225
IRIfs	16.56 (6.94)	17.24 (5.78)	−0.46	0.649
IRIec	18.90 (5.84)	17.21 (3.91)	1.41	0.162
TAS	45.21 (12.92)	54.00 (13.91)	−1.41	0.167

**Table 2:** Prediction of understanding, decision-making and justification score by control variables with binomial logistic regressions and mixed linear regressions.

	*b (SE)*	*F*	*p*
**SDS-17**			
Understanding	1.97 (0.47)	0.05	0.831
Decision-making	0.06 (0.05)	1.40	0.238
Justification	−0.01 (0.03)	0.29	0.590
**IRIpt**			
Understanding	0.02 (0.02)	0.79	0.374
Decision-making	0.01 (0.03)	0.13	0.723
Justification	0.00 (0.02)	0.01	0.910

*Each variable is tested individually and the group factor is not included in the analyses for this step. SDS-17, Social Desirability Scale -17; IRIpt, Interpersonal Reactivity Index – Perspective-taking; SE, standard error.*

### Group Differences in Moral Reasoning

**FIGURE 3:**
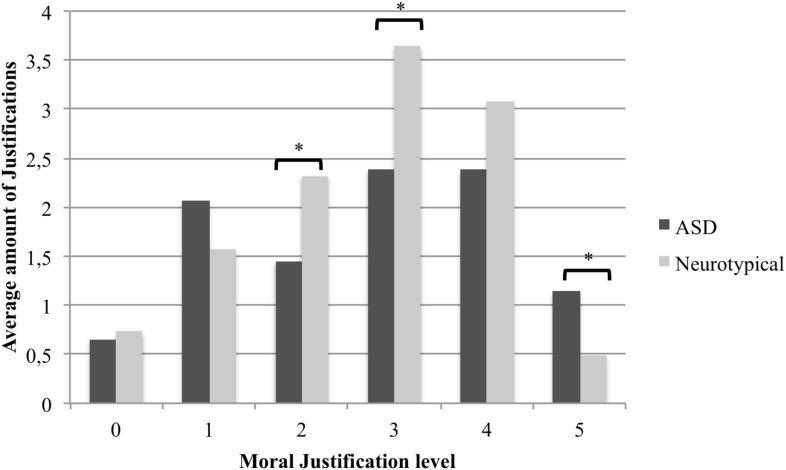
Comparison of control and ASD groups on Justification.

Understanding of the moral dilemmas was poorer in ASD (*M* = 11.79, *SD* = 2.54) compared to control participants (*M* = 13.94, *SD* = 1.73), *t*(84) = 4.63, *p* < 0.001. Significant differences were also found between groups on total moral Decision-making score, *t*(85) = 2.55, *p* = 0.012, with higher scores obtained by neurotypical controls (*M* = 12.38, *SD* = 2.19) compared to participant with ASD (*M* = 11.00, *SD* = 2.71). However, total moral Justification score were comparable across groups (control: *M* = 38.71, *SD* = 10.25, ASD: *M* = 33.86, *SD* = 14.15), *t*(85) = 1.82, *p* = 0.072. Comparisons assessing group differences in the types of answers provided by participants for each justification level individually (**Figure 3**) showed that participants with ASD produced a greater number of level-5 answers, whereas neurotypical participants had more level-2, and level-3 answers. Both groups were similar on their amount of level-0, -1, and -4 answers.

### Group Differences in Visual Encoding

Eye-tracking data were cleaned and 188 trials were dismissed (13.82%) because no TTFF was available. The proportion of lost data for the included trials was 18.25% (*SD* = 21.18), which is not uncommon for this model of eye-tracker (Holmqvist, 2017). Participants of both groups were comparable with regard to the average proportion of dismissed trials, *t*(87) = 1.37, *p* = 0.176, and the proportion of lost data *t*(87) = 1.01, *p* = 0.318. The average TTFF on faces in seconds was shorter for control participants (*M* = 0.70, *SD* = 0.26) compared to ASD participants (*M* = 0.91, *SD* = 0.21), *t*(81) = -3.54, *p* = 0.001. The results for FC were in the same direction, but did not reach the threshold of statistical significance (control: *M* = 6.44, *SD* = 2.61, ASD: *M* = 5.28, *SD* = 2.85), *t*(83) = 1.90, *p* = 0.062. Pupil dilation was similar across groups (control: *M* = 3.94, *SD* = 0.72, ASD: *M* = 4.23, *SD* = 0.80), *t*(87) = -1.70, *p* = 0.093.

### Visual Encoding and Moral Understanding

When accounting for ROI size, there was no effect of FC on moral Understanding, *b* = −0.02, *SE* = 0.03, OR at 95% CI = 0.92; 1.04, *p* = 0.437. There was no main effect of TTFF, *b* = −0.06, *SE* = 0.25, OR at 95% CI = 0.58; 1.53, *p* = 0.802, or Pupil dilation, *b* = 0.06, *SE* = 0.26, OR at 95% CI = 0.64; 1.77, *p* = 0.808, on moral Understanding. Similarly, no interaction between eye-tracking variables and group were statistically significant, *p*s ≥ 0.448. Of note, when the interactions were not included in the model, there was a main effect of Group; neurotypical participants understood more moral dilemmas than participants with ASD, *b* = 0.86, *SE* = 0.25, OR at 95% CI = 1.46; 3.82, *p* < 0.001.

### Visual Encoding and Moral Decision-Making

**Table 3** presents the results of the prediction of moral Decision-making by our variables of interest when controlling for ROI size for the understood dilemmas. TTFF was a significant predictor of moral Decision-making. This effect was similar for both groups, as no interaction between group and eye-tracking variables was significant. Otherwise, no main effects were found for Group, FC, or Pupil dilation (**Table 3**).

**Table 3:** Prediction of moral decision-making by Group, TTFF, FC, and Pupil dilation.

		95% CI for odds ratio			
	*b (SE)*	Lower	Odds ratio	Upper	*p*
Intercept	2.77 (1.86)	0.42	15.94	607.37	0.136
ROI size	0.02 (0.04)	0.95	1.02	1.11	0.577
Group (control)	−1.91 (2.06)	0.00	0.15	8.42	0.354
TTFF	−0.92^∗^ (0.46)	0.16	0.40	0.97	0.044
FC	−0.06 (0.06)	0.84	0.94	1.06	0.313
Pupil dilation	0.28 (0.40)	0.60	1.32	2.89	0.486
TTFF ^∗^ Group	0.68 (0.52)	0.71	1.97	5.42	0.192
FC ^∗^ Group	0.11 (0.07)	0.98	1.12	1.27	0.093
Pupil dilation ^∗^ Group	−0.0 (0.46)	0.40	1.00	2.46	0.993

*CS Covariance: b = 0.60, SE = 0.25, OR at 95% CI = 0.27;1.34, p = 0.015, ROI, region of interest; FC, fixation count; TTFF, time to first fixation; SE, standard error. ^∗^p < 0.05.*

### Visual Encoding and Moral Justification

**Table 4** presents the results of the prediction of moral Justification level by our variables of interest when controlling for ROI size for the understood dilemmas. A significant main effect of eye movement was found for FC (**Table 4**). However, no effect of Group, TTFF, Pupil dilation, or interaction was significant.

**Table 4:** Prediction of moral Justification by Group, TTFF, FC, and Pupil dilation.

	*b (SE)*	95% CI	*p*
Intercept	2.16^∗^ (0.78)	0.62; 3.70	0.007
ROI size	−0.04^∗^ (0.1)	−0.06; −0.01	0.006
Group (control)	1.08 (0.93)	−0.75; 2.91	0.246
TTFF	−0.06 (0.14)	−0.21; 0.33	0.657
FC	0.06^∗^ (0.02)	0.02; 0.09	0.001
Pupil dilation	0.11 (0.18)	−0.25; 0.47	0.552
TTFF ^∗^ Group	−0.20 (0.17)	−0.53; 0.14	0.245
FC ^∗^ Group	−0.02 (0.02)	−0.06; 0.01	0.217
Pupil dilation ^∗^ Group	−0.20 (0.22)	−0.64; 0.23	0.357

*Repeated measure CS covariance: b = 1.29, SE = 0.06, 95% CI = 1.17;1.41, p < 0.001, ROI, region of interest; FC, fixation count; TTFF, time to first fixation; SE, standard error. ^∗^p < 0.05.*

## Discussion

### Summary of Key Findings

The objective of this study was to explore the relation between visual encoding of social information and moral reasoning in ASD using an ecological visual paradigm. As expected, eye movements differed between groups during the presentation of moral dilemmas, with participants with ASD looking at faces later than their neurotypical counterparts. However, both groups produced a comparable amount of fixations toward social cues and showed similar sympathetic arousal, as reflected by pupil dilation. With respect to moral reasoning, participants with ASD had more difficulties understanding the dilemmas presented than controls and they produced fewer socially adaptive moral decisions. However, both groups were comparable in terms of average moral justification level. Interestingly, however, the pattern of justifications differed: participants with ASD produced more level-5 responses on the SoMoral task, while neurotypical participants produced more level-2 and level-3 responses. Given that level-5 responses are associated with an emphasis on universal principles, and level-2 and -3 with an emphasis on interpersonal exchange and social relationships, this finding corroborates our expectation of qualitative differences in moral justification (i.e., greater reliance on rules and fewer justifications oriented toward social relations in ASD) and is consistent with reduced reliance on perspective taking for moral judgment in ASD (Fadda et al., 2016), but did not result in a lower level of moral reasoning overall. In general, although the difference in the number of level-1 responses did not reach statistical significance, participants with ASD tended to produce more responses at the ends of the scoring spectrum (level-1 and 5), whereas neurotypical participants’ responses were more centered (level-2 and 3). As expected, there was a link between visual encoding measures and moral reasoning, but the relation between the two constructs was similar in ASD and controls. More precisely, increased attention on faces was associated with the production of higher-level justifications for both groups. Similarly, faster fixations directed toward faces were also associated with an increase in adaptive decision-making for both groups. As such, the similar fixation counts for both groups are associated with comparable average justification level. The increased time to first fixation in ASD is associated with a lower rate of adaptive moral decisions. These results suggest that visual encoding contributes to two aspects of moral reasoning, namely decision-making and the level of justification. However, the absence of interaction between eye-tracking variables and group indicates that the contribution of visual encoding to moral reasoning is similar in neurotypical participants and participants with ASD. In summary, individuals with ASD exhibited poorer understanding of moral dilemmas, produced fewer socially adaptive decisions and showed differences in the quality of moral justifications they provided to everyday moral dilemmas compared to neurotypical individuals. However, these differences appear to be attributable to differences in the visual encoding of social cues only for decision-making.

### Comparisons With Previous Research in Moral Reasoning and ASD

Many of the findings of this study are consistent with characteristics of ASD documented in previous work. First, eye-tracking measures showed visual encoding patterns typical of ASD individuals including taking a longer time to fixate on faces. Some particularities that characterize ASD in terms of dispositional variables were also expected, including poorer perspective-taking skills, consistent with studies reporting impaired theory of mind, but preserved affective empathy in ASD (Rogers et al., 2007; Dziobek et al., 2008). Participants with ASD were more prone to social desirability while the opposite pattern might have been expected (Izuma et al., 2011). This finding is, however, plausible when attention is paid to individual items on the scale. Indeed, some of the items may suggest socially desirability responding in neurotypical participants, but may rather be a reflection of atypical social life (i.e., “I have tried illegal drugs.”) or behaviors (i.e., “During arguments I always stay objective and matter-of-fact”; “When I have made a promise, I keep it–no ifs, ands or buts”) in ASD. Of note, however, these group differences were not related to any aspects of moral reasoning.

Unlike studies using verbal stimuli such as written dilemmas, participants with ASD in the present study showed reduced moral understanding. This is consistent with the hypothesis that the medium in which dilemmas are presented may be critical for the understanding of social situations and more particularly of moral issues. In this study, presenting ecological, visual stimuli to participants brought to light differences between ASD and control groups that may otherwise have remained undetected. However, although the type of paradigm may contribute to moral understanding, again no relation was found with visual encoding. Thus, differences in subsequent stages of social information processing (e.g., interpretation of cues, clarification of goal, response construction) may instead be key to explaining reduced moral understanding in ASD. For example, the interpretation stage, which immediately follows the encoding stage (Crick and Dodge, 1994; Lemerise and Arsenio, 2000), involves searching in long term memory for matching social scripts or social rules and conventions, attributing intent to others, evaluating others and self. Several of these abilities are likely to be altered in ASD. In sum, although speculative, the simplest and most plausible interpretation is that non-adaptive social behaviors may stem from a misinterpretation of social situations and not necessarily from changes in the encoding of social information.

### Methodological Implications of the Study Findings

Although this study was conducted in a laboratory and, as such, has limited generalizability to real life situations, it can reasonably be considered as a step forward in the measurement of moral reasoning in comparison with non-ecological paradigms. Using the SoMoral to measure moral reasoning brought to light the importance of visual social information processing in the production and the justification of moral decisions in situations representative of everyday life. More specifically, when social cues are processed as important information (earlier and more frequent gaze fixations), the probability of producing an adaptive decision and higher-level moral justification increased. The importance of these elements may be underestimated in the literature, as social information processing and moral reasoning have not typically been empirically investigated together (Arsenio and Lemerise, 2004; Dodge and Rabiner, 2004; Greenwood, 2011). More generally, this study also emphasizes the relevance of studying multiple aspects of moral reasoning (i.e., understanding, decision-making, justification) for a broader understanding of how the phenomenon is embodied in a social context. Though it may be partially constructed after decision-making, we found that the production of a justification is linked to early perceptual cognitive processes. Also, even if the relation between decision-making and justification is still unclear, the production of a relevant and convincing justification can contribute to social adjustment (Haidt and Bjorklund, 2008). It is thus important to consider the production of a convincing justification as an important part of real life moral reasoning.

### Clinical Implications of the Study Findings

The hypothesis of differential mechanisms underlying moral reasoning in ASD is partially supported by the results. First, the idea that individuals with ASD use different strategies involving idiosyncratic information processing when faced with moral dilemmas is supported by the behavioral data. According to the justifications provided by the participants, they appear to base their decisions on different modes of reasoning, as reflected by the patterns of reasoning levels. Neurotypical participants produced more justifications that were oriented toward pragmatic and egocentric exchange (level-2). These responses were characterized by gaining personal benefits and interactions with others for the purpose of mutual favors (e.g., “I would do it for him because he would do it for me”). They also produced more justifications that were characterized by a marked orientation toward interpersonal relationships and reputation management, including justifications based on interpersonal empathy and trust (level-3). Although they present obvious differences, these two types of reasoning have in common that they are mainly articulated around relationships with others, either for obtaining personal benefits, or for the maintenance of harmonious interpersonal bonds. In this sense, a decreased presence of this type of response is consistent with a lack of behavioral adjustment reported in individuals with ASD to fawn (Chevallier et al., 2012), mask stereotypes (Birmingham et al., 2015), or appear generous (Izuma et al., 2011).

Interestingly, participants with ASD produced more level-5 justifications, characterized by an evaluation of social contracts. At this level, there is a detachment from social norms and interpersonal relationships to focus on universal and fundamental moral principles. This result is coherent with the group difference in the importance given to social aspects of the moral dilemma. This justification level relegates the social or interpersonal aspects of dilemmas to the background, in favor of the protection of fundamental values and moral principles. In this sense, this type of reasoning is considered to be less flexible, a finding consistent with the observation that individuals with ASD may tend to exhibit cognitive and behavioral rigidity (Van Eylen et al., 2011; D’Cruz et al., 2013). It is interesting to note that, although it may be less frequently produced in everyday life by typically developing individuals, it is considered to be the highest level of moral justification, where social norms are challenged in favor of a pattern of reasoning oriented toward rational thinking.

The notion of a differential mechanism of moral reasoning in ASD is only partially supported by the study results, as the eye-tracking measures showed that the role played by visual encoding was comparable across both groups. This suggests that the cognitive mechanism linking visual encoding of social information and decision-making is similar in neurotypical participants and participants with ASD, suggesting that both groups use social cues in a similar way to make a decision in the context of a moral dilemma. Slower orientation toward these cues in participants with ASD may contribute to the smaller number of socially adaptive responses. Also, contrary to what has been proposed in the literature (De Vignemont and Frith, 2008; McGeer, 2008; Barnes et al., 2009; Brewer et al., 2015), participants with ASD did not make moral decisions at a level comparable to the control group. It is possible that previous studies using stimuli that are less ecological encourage the use of compensatory strategies based on language or reading skills, which was not possible in our methodology. In this sense, the use of real life scenarios presented visually highlighted differences in moral decision-making in ASD that may not have been detected otherwise. Nevertheless, it is also possible that an alternative information processing strategy underlies moral reasoning in ASD after the initial visual encoding. For example, differences in the interpretation of social cues or in the search in long-term memory for relevant social scripts could explain the differences in the justifications provided. It is also plausible that other socio-cognitive skills (e.g., theory of mind and empathy) contribute to moral processes in participants with ASD.

More broadly, the observed results also shed light on the social functioning of individuals with ASD. If they take more time to direct their attention toward faces, they may miss encoding opportunities in the fast interactions of everyday life and thus respond to them incorrectly. Moreover, even when they understand the moral issues at play, they make fewer appropriate decisions. Finally, they appear to rely on a type of reasoning that is less often evoked in the general population, which may detract from what is expected or accepted by others (especially in combination with a decision that is not socially adaptive). Each of these particularities may have a snowball effect in social functioning and could potentially underlie social consequences of a greater magnitude. Thus, the observations made in this study may be applicable to broader social contexts than only moral reasoning.

### Limitations

This study presents some limitations that must be taken into account when interpreting and generalizing the results. Although the moral reasoning task is picture-based, the formulation of a justification nonetheless relies on verbal expression skills to some extent. In previous studies using the SoMoral task, level of moral justification was found to correlate with verbal fluency (Vera-Estay et al., 2015). It is therefore possible that an individual with greater speaking fluency may be able to provide justifications of a higher level. However, the verbal IQ of participants in both groups was similar in this study. The use of a non-experimental design limits the scope of the results obtained in terms of causality; the relation between visual encoding and moral reasoning remains statistical only. While the presence of a relation between visual information encoding and moral reasoning is empirically demonstrated, the nature and significance of the contribution of visual encoding is not perfectly clear. A possibility remains that people who tend to provide more socially adaptive responses and to elaborate more mature justifications are also people who tend to pay more attention to social cues. Further research including experimental manipulation of the independent variables could help clarify this aspect.

## Conclusion

This study offers a new perspective on fundamental issues in the study of moral reasoning within ASD. Additional studies will be required to establish a comprehensive explanatory model of the relations between visual processing of social information and moral reasoning in ASD. While developmental studies indicate a linear progression in SoMoral justification score (Chiasson et al., 2017), certain patterns of responses would possibly be better indicators of the qualitative differences that characterize conditions such as ASD. An adequate understanding of those differences would provide more nuanced information about an individual’s moral reasoning, and could be more relevant in the context of clinical intervention. To this end, an important implication of the findings is that there are various ways to achieve moral decision-making. In a clinical setting, the use of a reasoning approach closer to the justifications produced by participants with ASD could be more intuitive for them. For example, social stories are very widely used (Goodman-Scott et al., 2016; Olçay-Gül and Tekin-Iftar, 2016), but often focus on interpersonal relationships. The use of arguments based on fixed rules or universal moral principles could make them more accessible to people with ASD, allowing them to produce moral behaviors without confronting their own values.

## Data Availability

The raw data supporting the conclusions of this manuscript will be made available by the authors, without undue reservation, to any qualified researcher.

## Author Contributions

MG, MB, and BFd’A conceived and planned the experiment. MG and BFd’A recruited participants. MG and ML were involved in acquisition of data. MG, ML, and EE processed the experimental data and performed the analysis. MG drafted the manuscript and designed the figures. MB supervised the project. All authors discussed the results and commented on the manuscript.

## Conflict of Interest Statement

The authors declare that the research was conducted in the absence of any commercial or financial relationships that could be construed as a potential conflict of interest.
